# Sustainable strategies for hospital wastewater treatment: bioremediation, phytoremediation, and hybrid approaches for emerging pollutants

**DOI:** 10.3389/fmicb.2025.1710583

**Published:** 2026-01-23

**Authors:** Shubhra Sharma, Divya Prakash, Swarnima Agnihotri

**Affiliations:** 1 School of Biotechnology and Life Sciences, Shobhit Institute of Engineering and Technology, (NAAC Accredited Grade “A”, Deemed to-be-University), Modipuram, Meerut, India; 2 Swedish Centre for Resource Recovery, Department of Resource Recovery and Building Technology, University of Borås, Borås, Sweden

**Keywords:** antibiotic resistance genes, bioremediation, emerging pollutants, hospital wastewater, microbial degradation, pharmaceutical contaminants, phytoremediation

## Abstract

Hospital wastewater (HWW) is a complex matrix of pharmaceutical residues, antibiotic resistance genes (ARGs), pathogens, and emerging contaminants that threaten public health and ecosystems. Conventional wastewater treatment plants (WWTPs) often fail to eliminate persistent compounds like carbamazepine and sulfamethoxazole, contributing to antimicrobial resistance and environmental toxicity. This review explores advanced treatment strategies with a focus on bioremediation and phytoremediation. Microbial approaches using bacteria, fungi, algae such as *Labrys portucalensis*, *Trametes versicolor*, and *Chlorella vulgaris* demonstrate degradation of pharmaceuticals and ARGs. Similarly, phytoremediation with species like *Typha angustifolia* and *Vetiveria zizanioides* supports on-site through rhizospheric uptake. Integrated systems combining membrane bioreactors (MBRs), advanced oxidation processes (AOPs), constructed wetlands (CWs), and microbial consortia offer enhanced removal efficiency and ARG reduction. While hybrid systems show strong potential, they face challenges such as high costs, difficulties in large-scale application, and limited regulation. Overall, this review highlights how integrating biological and technological methods provides a practical and sustainable path forward for treating hospital wastewater (HWW) and reducing its environmental and health impacts. A multidisciplinary, globally coordinated approach is essential for sustainable HWW management.

## 1 Introduction

Hospital effluent is acknowledged as a significant source of environmental contamination. It contains pharmaceuticals, pathogens, and antimicrobial resistance (AMR) determinants that endure conventional treatment procedures. It is imperative to address these discharges in order to advance global sustainability objectives, defend public health, and preserve ecosystem integrity under the One Health framework. Pharmaceuticals, though essential in medical and industrial applications, have the capability to severely impact aquatic ecosystems and human health when released into water bodies even at concentrations below therapeutic doses (Lakhani et al., 2022). Their chemical diversity and biological activity complicate uniform treatment strategies. After consumption, these compounds and their metabolites are excreted and enter various water sources, including surface water, groundwater, and drinking supplies. Hospitals contribute significantly towards pharmaceutical pollution by discharging polluted effluents directly into municipal sewers. Conventional treatment plants lack the capacity to remove many pharmaceutical residues effectively (Bijlsma et al., 2021; Khan A. H. et al., 2020; Khan et al., 2019). For instance, while compounds like paracetamol degrade efficiently, others like carbamazepine persist through standard biological treatment processes such as activated sludge systems (Al Qarni et al., 2016). Furthermore, treated wastewater reused for agriculture or aquifer recharge may still contain persistent antibiotics such as streptomycin and gentamicin, while macrolides like erythromycin, clarithromycin, and azithromycin are frequently detected in European surface waters (Felis et al., 2020). This inefficacy contributes not only to contamination of surface and drinking water but also to the environmental spread of antimicrobial resistance. Inadequate regulations and weak monitoring further worsen the issue (Aydin et al., 2019). HWW has also tested positive for SARS-CoV-2, often reflecting infection levels in surrounding populations. Unlike viruses such as hepatitis A, SARS-CoV-2 requires a significantly higher chlorine dose (10 mg·min/L) for disinfection. The presence of pharmaceuticals, antibiotic resistance genes (ARGs), resistant bacteria, and other difficult-to-treat compounds highlights the urgent need for advanced treatment strategies (Khan et al., 2023).

A review of over 1,000 studies reported 631 pharmaceuticals in the environment, including diclofenac, sulfamethoxazole, EE2, carbamazepine, and tetracycline, across 71 countries in surface water, wasteswater treatment plant (WWTPs) effluents, soil, and drinking water. Diclofenac was found in 50 countries and exceeded its predicted no-effect concentration (PNEC) in 12. EE2, a synthetic estrogen, feminized male fish at just 5–6 ng/L, with surface water levels above its PNEC (0.01 ng/L) in 28 countries (Aus der Beek et al., 2016). Urban and HWW are major sources, but pharmaceutical manufacturing, veterinary waste, aquaculture, and irrigation with contaminated water also contribute. Despite >123,000 entries in the measured environmental concentration (MEC) database, large data gaps remain in Africa, Eastern Europe, and developing Asia. Still, diclofenac, EE2, and 14 other pharmaceuticals were detected across all UN regions, underscoring the need for standardized monitoring, inclusion of veterinary sources, and mixture toxicity assessment (Aus der Beek et al., 2016).

A study in Turkey found that hospitals contributed 13% of antibiotic loads in summer and up to 28% in winter. However, removal efficiency of conventional systems dropped from 79% in summer to 36% in winter, leaving behind compounds like azithromycin at ecologically dangerous levels (Aydin et al., 2019). Another study analyzing wastewater from seven large hospitals revealed persistent concentrations of nine high-risk drugs, including diclofenac, ibuprofen, and carbamazepine. While advanced oxidation processes (AOPs) like ozonation and O₃-H₂O₂ showed promise for some compounds, others like ofloxacin and ibuprofen were less effectively removed. At the same time, biological systems like membrane bioreactors and artificial wetlands have been tested to see if they are capable of eliminating pharmaceuticals while also lowering the number of microbes. However, their overall effectiveness depends on the composition of the influent, the operational settings, and how well the microbial consortia can handle changes. Adding hydrogen peroxide to AOPs sometimes made the therapy less effective, which lowered the overall efficiency. These findings collectively emphasize the constant difficulties posed by pharmaceutical residues in hospital effluents and stress the need for integrated, cost-efficient, and location-specific treatment strategies, especially in areas with limited resources (Khan N. A. et al., 2020).

Global monitoring has identified an extensive amount of various drugs in water systems. The absence of standardized methodologies, insufficient evaluation of cumulative toxicities, and inadequate integration of veterinary and industrial data result in significant deficiencies in risk modeling. This review specifically addresses these gaps by analyzing HWW as a significant yet inadequately regulated source of pharmaceutical residues and ARGs. The objective is to summarize existing knowledge regarding their prevalence, treatment difficulties, and microbiological and bioremediation approaches, while highlighting research and policy strategies for sustainable management.

## 2 Sources and nature of pharmaceutical contaminants

Pharmaceuticals enter environment through hospital discharges, municipal and industrial effluents, veterinary wastes, and landfill leachates. Their persistence and biological activity contribute to ecological risks, including antimicrobial resistance and pathogen proliferation. The occurrence and diversity of these contaminants vary globally, reflecting differences in usage and treatment efficiency. Table 1 summarizes recent studies reporting pharmaceuticals, pathogens, and ARGs detected in water and wastewater across continents.

**Table 1:** Summary of pharmaceuticals, pathogens, and ARGs detected in global water and wastewater systems, with suspected sources (Ajibola and Zwiener, 2022; Hayden et al., 2022; Mastrángelo et al., 2022; Saaristo et al., 2024; Wilkinson et al., 2022; Yao et al., 2021).

Continent (Country)	Pharmaceuticals reported	Pathogens/ARGs detected	Suspected source(s)
Africa (Nigeria)	Ciprofloxacin Ofloxacin Azithromycin Tetracyclines Sulfonamides	Antibiotic residues in sludge alongside different ARGs	HWW enters WWTP sludge Makes land application a risk.
Asia (China)	Fluoroquinolones Sulfonamides Macrolides Tetracyclines	Broad suite of ARGs associated with antibiotics ARG abundance reported	HWW Discharge to municipal sewer and receiving waters.
North America (USA)	Azithromycin OTC drugs Prescription drugs	Pharmaceuticals as contaminants	Municipal WWTP influent Hospital discharges Reusing irrigation acts as exposure.
Europe (multi-country)	Diclofenac Carbamazepin Antidepressants	Co-occurrence of antibiotics and ARGs downstream of WWTPs and hospitals	Municipal WWTP effluents Hospital releases Pharmaceutical manufacturing
South America (Argentina)	Multiple PhAC groups detected antibiotics Analgesics/NSAIDs Cardiovascular drugs	Pharmaceuticals in water ARG indicators downstream of sewage inputs.	Municipal WWTP effluent Urban runoff Veterinary and agricultural outflow.
Oceania (Australia- Victoria region and catchments)	Fluoxetine Sertraline Venlafaxine	Pathogenic bacterial (e.g., *Enterobacterales*, *Mycobacteriales*, *Legionellales*) co-occurring with chemical contaminants	WWTP effluents, sewage-impacted floodwaters Urban and agricultural land release.

### 2.1 Pharmaceutical residue

Human and animal excretion is the primary means that pharmaceutical contaminants enter water bodies, and wastewater from these sources is sent to treatment facilities. Other sources include veterinary, industrial, and medical waste, aquaculture, and agricultural runoff. While some substances, such as acetaminophen and caffeine, are easily broken down, others, such as sulfamethoxazole, carbamazepine, and cotinine, are more resilient in aquatic environments (Costa et al., 2019). Supporting this, groundwater monitoring near a psychiatric facility in the Czech Republic detected carbamazepine contamination over 1 km downstream from the discharge site. The minimal removal was attributed more to dilution than degradation, underscoring the long-term threat of pharmaceutical leaching (Rozman et al., 2015).

In Nairobi, a study found that sulfamethoxazole concentrations in HWW reached as high as 20.6 μg/L, and the substance was still present in WWTP effluents and surface waters. Sulfonamides pose pervasive ecological risks due to their anionic and neutral speciation, which results in high mobility and resistance to adsorption and degradation (Ngigi et al., 2020). Pharmaceutical residues are frequently not entirely eliminated by WWTPs, which results in their dispersion into water, sediment, and other environmental matrices (Fernandes et al., 2020).

The global utilization of antidepressants has increased significantly. Individuals with mental health disorders are prescribed these medications. There has been a sharp increase in the consumption of antidepressants globally, driven by a surge in mental health disorders. The COVID-19 pandemic further intensified anxiety and depression worldwide, leading to an even greater reliance on these medications (Frangou et al., 2023; Tiger et al., 2024; Yavuz-Guzel et al., 2022). Their discharge into surface water can have a substantial impact on aquatic organisms. Reduced activity, altered behaviors, and physiological issues, such as cardiac or organ injury, have been observed in fish, snails, and other invertebrates that have been exposed to antidepressants (Singh et al., 2022). Fluoxetine and venlafaxine have the potential to disrupt neurological and hormonal functions in species such as zebrafish, and their effects may be transmitted down to future generations. The Risk Quotient (RQ) analysis, which indicates that values of ≥1 indicate a high ecological risk, demonstrated that compounds such as sertraline, citalopram, and bupropion are particularly detrimental, even after wastewater treatment. Consequently, it is imperative to develop more effective removal strategies (Singh et al., 2022).

### 2.2 Pathogens and ARGs

Pharmaceuticals and pathogenic organisms are concentrated in HWW, which is generated in surgical wards, laboratories, and patient care units. Hormones, radioactive tracers, heavy metals, and a diverse array of pathogens, such as bacteria, viruses, fungi, and parasites, are present in HWW in addition to active medicinal compounds. Recent research has demonstrated that hospital-derived clinical waste contains a diverse array of drug-resistant fungal species, including *Candida albicans, Fusarium* spp., and *Rhizopus stolonifer*. These species are characterized by their resistance to antifungal agents and significant enzymatic activity. These results underscore the exacerbated health and environmental hazards that result from the improper treatment of biomedical waste in healthcare environments. Researchers have identified resistant bacteria such as *Aeromonas*, *Kluyvera*, *E. coli*, *Staphylococcus aureus*, *Acinetobacter*, and *Enterococcus* in HWW. These organisms are notably resistant to antibiotics such as ciprofloxacin, chloramphenicol, aminoglycosides, and carbapenems, demonstrating multidrug resistance (MDR) (Kaur et al., 2020; Singh et al., 2022). Recent research suggests that wastewater from oncology and pathology clinics is particularly hazardous, exhibiting an ecotoxicity and pharmaceutical concentrations that are 2–3 times higher than those of typical hospital effluents. These samples remained toxic even when diluted and contained multidrug-resistant strains, such as *E. coli* and *S. aureus*, that were characterized by the sul2, tetA, ampC, and qnrA genes (Kaliakatsos et al., 2023). The diagnostic approach has undergone a significant transformation, transitioning from conventional culture methods to rapid molecular assays such as ELISA, multiplex PCR, and digital PCR. These assays offer a precise and timely identification of pathogens. The microbial diversity of HWW is subject to fluctuations in accordance with the level of hospital activity, with a climax observed during the day. The microbial community is primarily dominated by *Proteobacteria*, *Firmicutes*, *Bacteroidetes*, and anaerobic gut organisms such as *Clostridales* and *Bifidobacteriales*. Fungal pathogens (e.g., *Aspergillus*, *Candida*) and viral agents (e.g., adenovirus, rotavirus) are frequently identified. HWW is transformed into a reservoir for resistance that conventional WWTPs are unable to fully neutralize as a result of the release of antibiotics, which promotes horizontal gene transfer (HGT) and the spread of antibiotic resistance genes (ARGs) (Kaur et al., 2020).

### 2.3 Microplastics as ARG vectors

Microplastics (MPs), particularly those derived from polystyrene, PET, and polypropylene, have been recognized as emergent vectors for antibiotic resistance genes (ARGs). Biofilm formation is facilitated by their small size and porous surface, which creates an environment for ARG-carrying bacteria, including *Flavobacterium*, *Chryseobacterium*, and *Pseudomonas*. These MPs promote the co-selection and transfer of resistance characteristics through conjugation, transformation, and transduction by absorbing antibiotics and heavy metals. This process is also facilitated by biodegradable plastics such as PHA, though to a lesser extent. This issue gets even worse by hospital effluents, which are frequently contaminated with antimicrobials (Tuvo et al., 2023). This was observed in Italy, where carbapenem-resistant *E. coli* and *Acinetobacter* spp. were frequently reported (Tuvo et al., 2023). Despite the fact that tertiary treatments can eliminate up to 90% of MPs, millions continue to escape into ecosystems, where they accumulate in aquatic organisms, drinking water, and even human tissues. The consequences of this accumulation range from inflammation to cardiovascular risks (Tuvo et al., 2023). Varying capacities for biofilm formation were identified in a study of *Candida* spp. that was isolated from HWW. Strong biofilm-forming capabilities and resistance to antifungals such as itraconazole were demonstrated by strains such as *C. albicans-1* and *C. glabrata*. Sodium hypochlorite was generally effective against planktonic cells; however, it exhibited limited activity against biofilms unless subjected to protracted contact and high concentrations. The efficacy of benzalkonium chloride was even lower (Mataraci-Kara et al., 2020). These findings underscore the necessity of customized disinfection protocols and antifungal stewardship in the administration of healthcare wastewater (Mataraci-Kara et al., 2020).

### 2.4 Physico-chemical characteristics of HWW

Pharmaceutical concentrations in HWW are frequently 4–150 times higher than those in urban effluent (UWW). A study conducted at the SIPIBEL WWTP in France, which employs separate lines for HWW and UWW, demonstrated that the HWW effluent contained higher levels of nitrite, nitrate, and phosphate, despite the fact that both streams successfully removed organic matter and ammonium by over 90%. Paracetamol was nearly entirely eliminated (99.9%), while carbamazepine retained its high resistance (only 3.5% removal). Biofilm studies demonstrated that the microbial diversity of HWW-treated effluent (HTE) was substantially reduced in comparison to UWW-treated effluent (UTE), with community structure being significantly influenced by pharmaceutical content and seasonality. The impact of pharmaceutical pollutants on microbial ecology was underscored by the DNA fingerprinting that revealed UTE-associated biofilms had a higher species richness (Chonova et al., 2016).

A complex mixture of biological and chemical pollutants is present in hospital effluents. BOD₅, COD, and TSS levels are two to three times higher in physico-chemical assessments than in municipal effluent. Cytostatic medications, which are employed in chemotherapy, are a critical category of micropollutants that are frequently disregarded in conventional monitoring. In hospital effluents at concentrations exceeding 4,000 ng/L, a Spanish study identified high-risk compounds, including ifosfamide, imatinib, and methotrexate. Irinotecan and tamoxifen were found to be highly ranked on both the Hazard Quotient and PBT indices. Conventional treatment systems were unable to effectively remove these residues, resulting in hospital effluent being up to 15 times more toxic than municipal sewage (Olalla et al., 2018).

Trace metals, such as gadolinium and platinum, which are derived from chemotherapies and contrast agents, have been detected at concentrations of up to 300 μg/L. Over 40% of pharmaceutical residues are composed of analgesics, antibacterials, and anti-infectives, while psychiatric and oncology medications, such as cytostatics and antiepileptics, are also observed. Due to their ecological impact and persistence, compounds such as erythromycin and 17α-ethinylestradiol are identified on contaminant watchlists. Pharmaceutical loads range from 1.5 to 310 g/day, and HWW discharge can reach 570 m^3^/day. Due to their chemical stability and minimal biodegradability, the majority of WWTPs are not equipped to manage these micropollutants (Oliveira et al., 2018). WHO guidelines emphasize the necessity of pre-treatment protocols, particularly for departments that handle high-risk pharmaceuticals, and recommend a removal rate of over 95% of bacteria, as well as specific limits for helminths and toxic residues (Oliveira et al., 2018).

### 2.5 Classification of pharmaceutical micropollutants based on chemical stability and degradability

Pharmaceutical micropollutants exhibit varying degrees of persistence and reactivity in aquatic environments, depending upon their molecular structure and physicochemical characteristics. Antiepileptics like carbamazepine are recognized for their remarkable persistence, often found in treated effluents and surface waters. Their polycyclic aromatic structure and minimal reactivity with common oxidants provide exceptional chemical stability, while poor sorption characteristics enable them to remain predominantly dissolved and mobile. As a result, carbamazepine frequently endures standard treatment methods, necessitating AOPs, photocatalysis, adsorption on tailored carbons, or membrane polishing for efficient breakdown (Mestre and Carvalho, 2019). Mestre and Carvalho (2019) examined photocatalytic approaches aimed for persistent antiepileptics, highlighting the environmental resilience of carbamazepine across several treatment systems. Nonsteroidal anti-inflammatory medications (NSAIDs), such as diclofenac, ibuprofen, and naproxen, along with antibiotics like sulfonamides and fluoroquinolones, demonstrate diverse degradation characteristics. Ibuprofen is often efficiently eliminated through standard biological treatment; however, diclofenac exhibits significant persistence due to its electron-rich aromatic rings that inhibit biodegradation and only undergo partial photolysis in natural sunlight.

Alessandretti et al. (2021) identified diclofenac as one of the most commonly found NSAIDs in effluents, with improved AOPs and adsorption-based tertiary treatments demonstrating potential for its removal. Sulfonamides, including sulfamethoxazole, have moderate persistence; they are biodegradable under optimal conditions yet resistant to complete mineralization. Their degradation efficiency enhances by ozonation, peroxone, or integrated photocatalytic advanced oxidation processes (Martini et al., 2024). Conversely, fluoroquinolones such as ciprofloxacin and ofloxacin demonstrate significant sorption to sludge owing to metal chelation and zwitterionic characteristics, leading to accumulation in biosolids and inadequate removal in wastewater treatment facilities. Their removal generally necessitates integrated treatment systems that incorporate advanced oxidation processes, adsorption, and membrane separation, especially in hospital and industrial effluents (Huang et al., 2021).

## 3 Conventional treatment methods and their limitations

HWW comprises a complex mixture of pharmaceuticals, disinfectants, chemicals, and pathogens. Treatment typically begins with source-based waste categorization and disinfection using incineration, chemical disinfectants (e.g., sodium hypochlorite), or high-temperature steam. Incineration, employing rotary kilns or pyrolysis, can reduce waste volume by 90%, whereas chemical and steam methods are effective for smaller waste volumes (Wang et al., 2020).

### 3.1 Performance of conventional WWTPs

Conventional WWTPs usually rely on primary sedimentation, secondary biological processes (e.g., activated sludge), and tertiary disinfection. However, multiple studies have highlighted their inefficiency in completely removing pharmaceutical contaminants and resistant pathogens. For instance, Ganji et al. (2024) evaluated Zahedan’s WWTP and found that although microbial quality met agricultural reuse standards, COD (171 mg/L) and BOD₅ (44.4 mg/L) levels exceeded discharge limits. While treated sludge was agriculturally viable, the study recommended nanofiltration and activated carbon adsorption to further reduce heavy metals.

Across developing regions like Iran, Pakistan, and Bangladesh, Conventional activated sludge (CAS) systems remain dominant, often supplemented with sand filtration or chlorination. While TSS and COD removal exceeds 90%, persistent contaminants like *E. coli* and *Enterococci* sp. are commonly found in effluents (Al Aukidy et al., 2018). In Vietnam, a pilot MBR-NF system achieved over 93% COD removal and >4 LRV for coliforms, with 98–99% removal of iron and color. This underscores the advantage of hybrid systems in handling high organic loads and ensuring pathogen reduction (Tran et al., 2019).

### 3.2 Advanced oxidation processes

AOPs such as Fenton, UV/H₂O₂, ozonation, and photocatalysis generate hydroxyl radicals to degrade recalcitrant pollutants. Segura et al. (2021) found intensified Fenton oxidation most effective, removing 99.8% of 79 pharmaceuticals with the lowest hazard quotient (ΣHQ = 5.4), while catalytic wet air oxidation (CWAO) lagged in removing acetaminophen and ciprofloxacin. Nabavi et al. (2023) tested catalytic granular activated carbon treatment with UV (CGCT + UV) for amoxicillin removal, achieving 67.4% COD and 56.7% BOD reduction. However, CGCT + UV fell short of discharge standards, reinforcing the need for integration with biological treatments.

**Figure 1:**
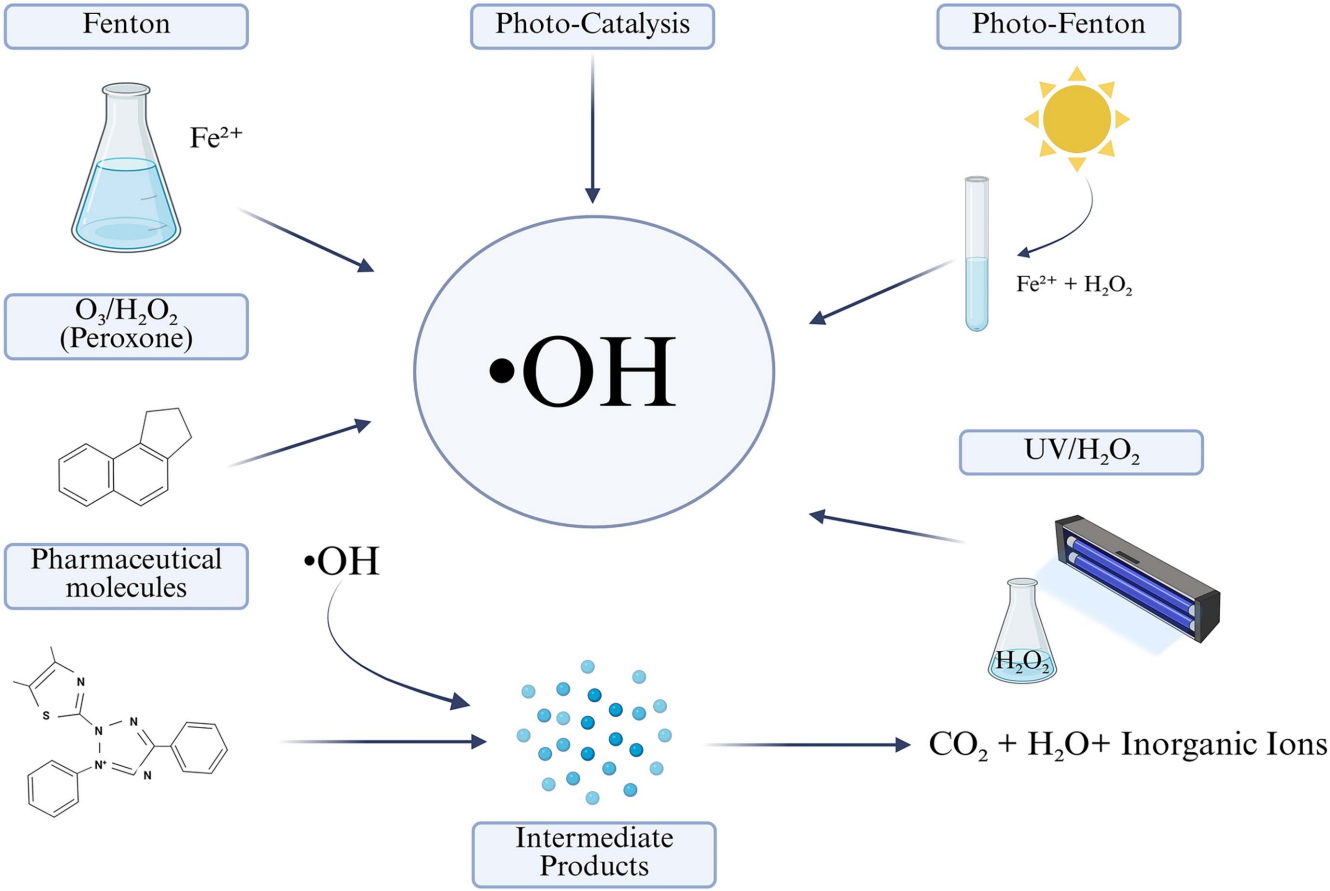
Overview of AOPs for pharmaceutical degradation.

As illustrated in Figure 1, hydroxyl radical (•OH) are generated through various AOPs such as Fenton, photo-Fenton, UV/H_2_O_2_ and photocatalysis.

- Generation of Hydroxyl Radicals (•OH):

Hydroxyl radicals are the main oxidizing agents responsible for breaking down pharmaceuticals. They can be produced by several AOP methods:

- Fenton Reaction (Fe^2+^/H_2_O_2_ system):

Fe^2+^ + H_2_O_2_ → Fe^3+^ + •OH + OH^−^

Photo-Fenton Reaction (UV-assisted):

Fe^3+^ + H_2_O + *hν* → Fe^2+^ + •OH + H^+^

Ozone and Hydrogen Peroxide (Peroxone Process):

O_3_ + H_2_ → O_2_•OH + O_2_ + HO_2_

UV/H_2_O_2_ Photolysis:

H_2_O_2_ + *hν* → 2•OH

Oxidation of Pharmaceutical Compounds:

Once hydroxyl radicals are formed, they attack the pharmaceutical molecules (RH) by abstraction or addition, leading to their degradation.

•OH + RH → R• + H2O
R• + O2 →ROO• → CO2 + H2O

- Simplified Reaction:

Pharmaceutical (organic compound) + •OH → CO_2_ + H_2_O + Inorganic ions.

Despite high efficiency, MBRs and AOPs often face cost and operational challenges. Even advanced systems leave behind micro-level contaminants. Beier et al. (2011) noted μg/L levels of diclofenac and carbamazepine in MBR effluent. Wiest et al. (2018) found sulfamethoxazole in treated hospital effluent at 810 ng/L, far above environmental safety thresholds (Afonso-Olivares et al., 2017; Garcia-Ivars et al., 2017; Guedes-Alonso et al., 2020). Confirmed reverse osmosis and nanofiltration outperform biological treatments in removing persistent pharmaceuticals like ofloxacin and diclofenac, though biological processes are more cost-effective for bulk organics. Cristóvão et al. (2019) validated Desal 5DK nanofilters as most effective for anticancer drug removal. Ajibola and Zwiener (2022) highlighted the persistence of ciprofloxacin and azithromycin in Nigerian sludge, warning against land application due to ecological risk.

The comparative performance of conventional and advanced HWW treatment systems is summarized in Table 2.

**Table 2:** Comparative overview of conventional and advanced HWW treatment (Nabavi et al., 2023; Pandis et al., 2022; Segura et al., 2021).

Aspects	Conventional treatment	Advanced treatment
Preliminary Treatment	Screening, grit removal	Screening, grit removal
Primary Treatment	Sedimentation	Sedimentation
Secondary Treatment	Activated Sludge or trickling filters	Biological treatment with Activated Sludge, MBR (Membrane Bioreactor), MBBR (Moving Bed Biofilm Reactor)
Tertiary/Advanced Processes	Rarely included	Membrane filtration, Advanced oxidation, Adsorption, Disinfection
Pharmaceutical Removal	Ineffective- drugs and antibiotics persist	Removes pharmaceutical residues and antibiotic-resistant genes
Heavy Metal Removal	Minimal	Possible with advanced adsorption and filtration
Pathogen Removal	Partial removal, many resistant microbes survive	High efficiency, elimates resistant bacteria and viruses
Final Effluent	Discharged into sewage or environment	Safe for reuse in non-potable application or safe discharge
Cost & Maintenance	Lower cost, easy to run, but less effective	Higher cost, requires skilled operation, but highly effective
Overall Limitation/Advantage	Incomplete removal of pharmaceutical and resistant pathogens	Comprehensive removal, address environmental and health risks

### 3.3 Biological treatment approaches

CAS systems often fail to eliminate persistent drugs like carbamazepine. Jaén-Gil et al. (2021) found that while UV/H₂O₂ was effective in pure water, real HWW reduced its efficacy. Combining AOP with CAS reduced both parent compounds and toxic transformation products of metoprolol and its metabolites more effectively than fungal treatments (Jaén-Gil et al., 2021).

Anaerobic and aerobic systems offer further solutions. Suresh and Abraham (2018) reported that Anaerobic Fixed Film Reactors (AFFRs) removed up to 90% of BOD, TSS, and TDS, and supported degradation of hormones like estradiol through microbial enzymes (e.g., monooxygenases, laccases, and proteases). Membrane Bioreactors (MBRs), used in German hospitals, effectively removed most pharmaceuticals, though compounds like carbamazepine persisted. Long sludge retention times (>100 days) improved biodegradation, but post-treatment polishing using ozonation or reverse osmosis remained essential (Beier et al., 2011).

### 3.4 Thermal and emerging oxidative techniques

Supercritical Water Oxidation (SCWO) offers compact, high-efficiency treatment. Top et al. Cy et al. (2020) achieved >90% removal of organic pollutants and >99% degradation of drugs like carbamazepine and cyclophosphamide at 450 °C and 25 MPa. The system required no additional post-treatment and degraded most contaminants within 60 s (Top et al., 2020). During this procedure, wastewater and an oxidant (hydrogen peroxide) are individually pumped, preheated, and combined at the reactor intake under supercritical conditions, wherein water exists as a singular phase with increased solubility for organic molecules and restricted solubility for inorganic salts. The organic contaminants are swiftly oxidized by the oxygen produced from hydrogen peroxide decomposition, transforming them into carbon dioxide, water, and mineral salts within seconds. The processed effluent is subsequently chilled, filtered, and depressurized prior to collection (Top et al., 2020).

### 3.5 Constructed wetlands and COVID-era advances

Constructed Wetlands (CWs) have gained traction due to their eco-friendliness. Liu et al. (2023) described CWs using substrates like LECA and alum sludge and plants like sugarcane to remove pharmaceuticals and ARGs via adsorption, hydrolysis, and rhizodegradation. Multi-stage CWs combined with MBRs offer modular, sustainable HWW treatment. COVID-19 emphasized viral risks in HWW. Activated Sludge Processes (ASPs) failed to remove viruses like SARS-CoV-2, while MBRs and AOPs (e.g., electroperoxone) showed higher efficacy. CWs proved scalable for decentralized applications and outbreak surveillance.

## 4 Bioremediation: principles, mechanisms, and agents

Pharmaceutical industry wastewater (PIWW) contains antibiotics, active drug residues, heavy metals, and microplastics posing severe environmental and health risks (Shah and Shah, 2020). Conventional treatments such as AOPs and physicochemical processes often fail to remove such pollutants completely, leading to rising interest in bioremediation. Bioremediation harnesses microorganisms to convert contaminants into less toxic compounds. This includes processes like biodegradation, biosorption, and enzymatic oxidation. Strategies vary based on pollutant type, contamination depth, and regulatory frameworks (Azubuike et al., 2016; Lakhani et al., 2022).

The rise of microbial biotechnology across agriculture, industry, and environmental sectors reflects its growing role in sustainable development. Applications range from rhizobial inoculants and algae-based water monitoring to bioremediation of polluted sites positioning microbes as key agents of ecological resilience (Ranjit et al., 2021).

### 4.1 Bacterial bioremediation

Microorganisms from activated sludge have shown high removal efficiencies for COD and heavy metals. *Aeromonas caviae, Moraxella osloensis,* and *Sphingobacterium thalpophilum* achieved over 70% COD reduction while also demonstrating Cr^6+^ biosorption, dye degradation, and oil deemulsification (A. H. Khan et al., 2020). Yeasts like *Galactomyces pseudocandidum* and *Rhodotorula mucilaginosa* also reached >70% COD removal (Khan A. H. et al., 2020). Bacterial strains like *Pseudomonas aeruginosa* and *Bacillus subtilis* effectively reduced lead, cadmium, chromium, and zinc levels in wastewater from Nigeria’s Wupa WWTP, bringing them within WHO discharge limits. Interestingly, single strains sometimes outperformed consortia for specific metals (Umar Faruok et al., 2024).

Multiple bacterial species such as *Comamonas aquatica, Bacillus* sp., *Rhizobium* sp. *C12* have been studied and it’ been reported that they have a significant role in degradation of NSAID like ibuprofen, diclofenac, carbamazepine and naproxen (Chakraborty et al., 2025). Var*iovorax* sp. degrades ibuprofen by monooxygenase-facilitated hydroxylation, followed by catechol-mediated ring cleavage and total mineralization. It is one of the few bacteria demonstrated to utilize ibuprofen as its exclusive carbon and energy source, rendering it a significant contributor to pharmaceutical bioremediation consortia (CY et al., 2020).

*Labrys portucalensis F11* degraded fluoroquinolones like ciprofloxacin and ofloxacin, achieving up to 91% removal via hydroxylation and defluorination. This strain also removed 99% of carbamazepine, forming transformation products such as Iminostilbene and Epoxy-CBZ (Amorim et al., 2014; Bessa et al., 2019). *Brevibacterium* sp. D4 degraded 90% of diclofenac under co-metabolism with acetate (Bessa et al., 2019). A study in Indonesia highlighted the use of indigenous hydrolytic bacteria isolated from hospital waste reservoirs for the treatment of liquid biomedical waste. These non-pathogenic strains, exhibiting robust lipase, protease, and amylase activity, achieved significant reductions in COD, BOD, TSS, and phosphate concentrations. The development of encapsulated bacterial consortia offers a WHO-aligned, cost-effective alternative to incineration in resource-limited settings (Ethica et al., 2018). Complementing this, a study from Lagos, Nigeria, found that heavy metals like cobalt, cadmium, and lead in HWW sediments significantly disrupted microbial diversity wiping out over 70,000 taxa while selecting for metal-tolerant bacteria and fungi such as *Laceyella sacchari*, *Aspergillus fumigatus*, and *Penicillium oxalicum*. These resilient strains, identified through metagenomic sequencing, may be suitable candidates for bioreactor-based remediation in heavy metal-rich, low-pH environments (Ogwugwa et al., 2021). *Bacillus paramycoides* and *Alcaligenes faecalis* strains isolated in Pakistan achieved over 90% reduction in BOD₅, COD, and pharmaceutical pollutants including phenol, caffeine, and diazepam while tolerating high heavy metal concentrations, making them ideal for dual bioremediation (Rashid et al., 2022).

### 4.2 Fungal bioremediation

Filamentous fungi, especially *Fusarium udum* and *F. solani*, reduced COD by 89–90%, outperforming bacteria and yeasts in activated sludge (Rozitis and Strade, 2015). Their efficacy stems from high laccase activity and tolerance to toxic substrates. *Trametes versicolor*, a white-rot fungus, has emerged as a lead candidate for removing ibuprofen, ketoprofen, and even ARGs from hospital effluents. Pretreatment with coagulation and UV-C, followed by continuous bioreactor operation, kept fungal pellets viable for 28 days and achieved >80% ibuprofen removal (Mir-Tutusaus et al., 2016). Further reinforcing its utility, a study using *Trametes versicolor* in a fluidized-bed bioreactor reported ≥99% removal of analgesics like diclofenac and ibuprofen, along with over 94% overall degradation of PhACs and EDCs under sterile conditions. Though efficacy dropped in non-sterile samples due to bacterial interference, high laccase activity and a 66% reduction in effluent toxicity confirmed the fungus’s strong metabolic performance (Cruz-Morató et al., 2014).

Fungal-bacterial biofilms on wooden rotating biological contactors removed >80% of PhACs including azithromycin and metronidazole without external aeration or reinoculation (Del Álamo et al., 2022). In a long-term bioreactor study treating psychiatric HWW, *Trametes versicolor* maintained dominance in both pellet and liquid matrices, achieving up to 80% pharmaceutical removal including 11 psychiatric drugs through enzymatic degradation involving both laccase and cytochrome P450 enzymes. Despite a decline in laccase activity after day 63, reactor efficacy remained stable due to microbial synergy, although reaccumulation of compounds like carbamazepine and venlafaxine highlighted the need for transformation product monitoring and microbial community management (Mir-Tutusaus et al., 2019). In a similar approach, *Penicillium oxalicum XD-3.1* was used to treat non-sterile HWW and demonstrated efficient removal of NSAIDs, paracetamol, and carbamazepine. Beyond its degradative capacity, the fungus suppressed native pathogenic microbes and reshaped the bacterial community structure acting as both a biocatalyst and microbial modulator (Olicón-Hernández et al., 2021). In non-sterile bioreactors, *T. versicolor* initially dominated, but later degradation was driven by microbial consortia involving *Candida*, *Fusarium*, and *Burkholderia* (Badia-Fabregat et al., 2016).

HWW was found to contain 45 pharmaceutical active compounds, with significantly higher concentrations of antibiotics, analgesics, and antihypertensives compared to municipal effluent. Among tested biological agents, *Trametes versicolor* achieved the highest removal efficiency and reduced environmental hazard quotients by nearly 50%, making it a leading candidate for bioremediation of hospital effluents (Álamo et al., 2021). Another study showed *T. versicolor* reduced 77% of antibiotics and ARGs like ermB and blaTEM in veterinary wastewater, altering microbial community structure and reducing selective pressure (Lucas et al., 2016).

### 4.3 Algal and Phycoremediation

Algae remove pharmaceuticals mainly through oxidative and enzymatic reactions driven by light. During photosynthesis, they produce reactive oxygen species like singlet oxygen and hydroxyl radicals that attack and break down drug molecules. Inside the cells, enzymes such as laccases, peroxidases, and monooxygenases further transform these oxidized compounds into simpler, less toxic metabolites. This combination of photochemical oxidation and enzymatic biotransformation makes algae an efficient tool for degrading persistent pharmaceuticals in wastewater (Chojnacka et al., 2022).

*Chlorella sorokiniana* and *Dunaliella tertiolecta* have shown strong potential for removing macrolide and fluoroquinolone antibiotics such as azithromycin and ofloxacin. *C. sorokiniana* primarily degrades azithromycin through enzymatic biodegradation supported by light-induced reactive oxygen species, while *D. tertiolecta* facilitates ofloxacin removal mainly via photodegradation, where hydroxyl radicals generated during photosynthesis initiate molecular breakdown (Hom-Diaz et al., 2022).

Algae like *Chlorella vulgaris*, *Nannochloris*, and *Scenedesmus obliquus* remove antibiotics such as triclosan, erythromycin, and sulfamethoxazole through biosorption, photolysis, and biodegradation. Algal turf scrubbers and photobioreactors enhance scalability (Guleri et al., 2020). *Chlorella pyrenoidosa* and genetically modified strains like PY-ZU1 can eliminate PhACs like ethinylestradiol via enzymatic degradation and bioaccumulation (Srinivasan et al., 2025).

### 4.4 Hybrid and electro-microbial systems

Microbial Fuel Cells (MFCs) and Microbial Electrochemical Technologies (METs) use electroactive bacteria like *Pseudomonas* and *Firmicutes* to degrade pollutants while generating electricity. Up to 85% metronidazole removal has been achieved within 24 h (Saeed et al., 2024). Another study demonstrated the potential of a fungal-based microbial fuel cell (FMFC) using *Trichoderma harzianum* and *Trametes trogii* for simultaneous degradation of organic pollutants and energy generation. Four compounds acetaminophen (APAP), para-aminophenol (PAP), methylene blue (MB), and sulfanilamide (SFA) were effectively degraded following pseudo-first-order kinetics (Gorin et al., 2024).

A constructed wetland-microbial fuel cell (CW-MFC) planted with *Eichhornia crassipes* was used to remove ibuprofen while generating electricity. Higher ibuprofen levels initially reduced microbial activity, but the system recovered as plant roots enhanced microbial metabolism and electron transfer. The findings suggest that integrating plants into microbial fuel cells can improve pharmaceutical degradation and boost bioelectricity generation (Youssef et al., 2023).

### 4.5 Enzyme-driven bioremediation

Laccases are multicopper oxidase enzymes that facilitate the oxidation of phenolic and non-phenolic aromatic compounds by transferring electrons from the substrate to molecular oxygen, resulting in the formation of water. Extracellular laccase from *T. versicolor* has demonstrated the capacity to breakdown pharmaceutical substances, specifically Diclofenac and Sulfamethoxazole. The laccase oxidizes the aromatic ring or side-chain of these medications, generating reactive radical intermediates that participate in subsequent non-enzymatic processes (Litwińska et al., 2019).

Another enzyme cytochrome P450s enzymes are natural catalysts that help microbes break down complex drugs by adding oxygen to them. They attach to the pharmaceutical molecule, activate oxygen, and insert it into the structure often forming a hydroxyl or epoxide group. This small chemical change makes the compound more water-soluble and easier for other enzymes or reactions to further break down into harmless forms (Kariyawasam et al., 2024).

Hydroxylases and demethylases play key roles in the early breakdown of pharmaceuticals. Hydroxylases add hydroxyl (–OH) groups to aromatic rings or side chains, while demethylases remove methyl (–CH₃) groups, making drug molecules more polar and easier for microbes to degrade further (Chen et al., 2022).

Immobilization techniques like alginate-silica-PVA beads and nanofibers enhance enzyme stability and reuse. Pd-AGS systems using palladium nanoparticles further amplify removal efficiency (Wojcieszyńska et al., 2023).

Table 3 summarizes various organism-based bioremediation approaches employed for the removal of pharmaceutical contaminants. It highlights the microorganisms commonly used and the specific pharmaceuticals targeted in different studies.

**Table 3:** Organism-based bioremediation approaches for pharmaceutical contaminants (Chmelová et al., 2024; Li et al., 2022; Mohamed et al., 2023; Pápai et al., 2023).

Organism/Enzyme	Target pollutant(s)	Mechanism
*Trametes versicolor*	NSAIDs (ibuprofen, ketoprofen), some antibiotics, hormones	Extracellular oxidoreductases, direct oxidation and LMS (laccase-mediator systems).
*Chlorella* sp., *Scenedesmus* sp.	Sulfamethoxazole, ofloxacin, fluoroquinolones	Biosorption, bioaccumulation, biodegradation (algal oxidoreductases)
*Pseudomonas* sp., *Rhodococcus* sp., *Bacillus* sp.	Carbamazepine, diclofenac, tetracyclines	Biodegradation via mono/di-oxygenases, hydrolases, reductases
*Nocardioides* sp.	Ibuprofen & Carbamazepine	Cometabolic biodegradation
*Alcaligenes aquatlilis (S2)*	Diclofenac	Aromatic hydroxylation, decarboxylation

Table 4 presents the major advantages and limitations of bioremediation, outlining its environmental benefits, cost-effectiveness, and sustainability.

**Table 4:** Advantages and limitations of bioremediation (Abatenh et al., 2017; Sharma, 2020; Sharma, 2019).

Advantages	Limitations
Eco-friendly, cost-effective, and scalable	Only works for biodegradable
Products non-toxic end products (CO_2,_ H_2_O)	Requires optimal conditions (pH, temp, nutrients)
Minimal manual intervention	Requires more time than conventional methods
Suitable for in-situ applications	Difficult to scale from lab to field
Can restore long-term environmental balance	Mixed contaminants pose treatment challenges

## 5 Phytoremediation: principles, mechanisms, and plant species

Phytoremediation is an eco-friendly, plant-based technology used to clean contaminated water, soil, and air by leveraging the biological activity of plants and their associated microbes. Also called green or vegetative remediation, it employs strategies like phytoextraction, phytostabilization, rhizodegradation, and phytovolatilization depending on the nature of the contaminant and the environment (Lakhani et al., 2022).

### 5.1 Relevance to HWW

HWW is a complex matrix containing pharmaceutical residues, disinfectants, pathogens, radioactive compounds, and heavy metals. Despite its toxicity, many countries lack clear guidelines for hospital effluent treatment. For instance, EU Directive 91/271/CEE overlooks hospital-specific discharge norms, while WHO only provides general recommendations. France’s Bellecombe pilot site exemplifies successful decentralized HWW treatment via collaboration and (Carraro et al., 2018).

Phytoremediation is especially valuable in regions lacking advanced wastewater infrastructure. Its integration with microbial communities in the rhizosphere boosts soil health and pollutant bioavailability. Root exudates enhance microbial degradation by stimulating enzymatic activity and organic matter turnover (Ghany et al., 2015).

### 5.2 Mechanisms of phytoremediation

**Figure 2:**
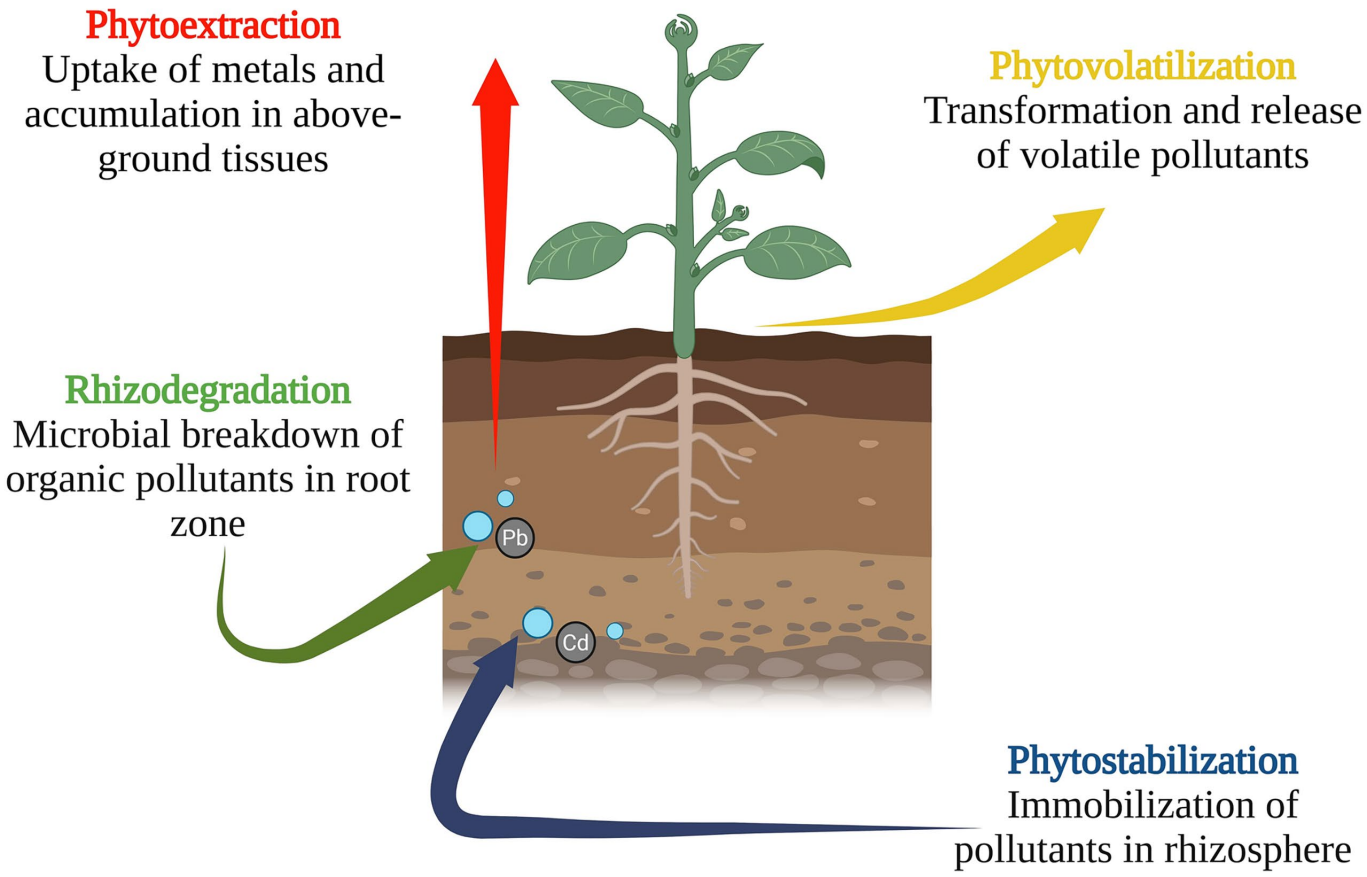
Multiple mechanisms used in phytoremediation.

Plants remediate pollutants via multiple mechanisms depicted in Figure 2.

Monocot grasses with dense root systems like *Vetiveria zizanioides* are particularly effective due to their soil-penetrating ability and ability to activate pollutant-degrading microbes (Khan et al., 2023; Ghany et al., 2015).

### 5.3 Evidence from aquatic and terrestrial plants

A study using *Typha angustifolia* (cattail) and *Eichhornia crassipes* (water hyacinth) to treat wastewater from Thai markets found that cattail outperformed hyacinth in biomass production and removal of BOD (96.2%), NH₃-N (96.8%), and TKN (79.2%). Metal uptake (Zn > Cd > Pb) was highest in roots, suggesting phytostabilization as the main mechanism. Additionally, *T. angustifolia* was superior for bioenergy due to its higher cellulose and lower ash content (Sricoth et al., 2018).

*Ipomoea aquatica* (water spinach) showed strong phytoremediation potential for Pb and Cr from HWW, adapting well to changing oxygen and pH levels. Though effective, extended exposure led to leaf yellowing, indicating plant stress (Suherman et al., 2021).

Experimental investigations have confirmed the capability of *Brassica juncea* (Indian mustard) to accumulate heavy metals in contaminated soils. When cultivated in soil with variable concentrations of nickel (Ni), chromium (Cr), lead (Pb), cadmium (Cd), and mercury (Hg), the plant demonstrated significant uptake of most metals, with the highest accumulation observed in the leaves (Sheetal et al., 2016).

Cyanobacteria like *Phormidium fragile* also play a role. Under optimized conditions, it achieved complete removal of ammonia and phosphorus from lab effluents and showed significant reductions in BOD and COD from pharmaceutical manufacturing wastewater. Pre-starvation of cyanobacteria further enhanced treatment efficiency (Lakhani et al., 2022).

Some promising plant species utilized in phytoremediation for the removal of pharmaceutical contaminants, along with their specific applications and mechanisms involved in contaminant uptake and degradation are listed in Table 5.

**Table 5:** Promising plant species and phytopharmaceutical applications (Kochi et al., 2023; Mumtaj et al., 2025; Panja et al., 2021; Ramirez-Muñoz et al., 2025; Shikha and Gauba, 2016; Srinivasan and Srivastava, 2024; Verma et al., 2024; Zapata-Morales et al., 2023).

Plant species	Target pollutants	Mechanism
*Typha latifolia*	Diclofenac and naproxen	Adsorption
*Helianthus annuus*	Cd, Pb, Ni, Zn	Phytoextraction
*Thlaspi caerulescens*	Cd, Zn	Hyperaccumulation
*Eichhornia crassipes*	Naproxen & ciprofloxacin	Adsorption
*Lemna minor*	Ciprofloxacin	Enzymatic detoxification
*Salvinia molesta*	Sulfamethoxazole	Enzymatic detoxification
*Helianthus annuus*	Carbamazepine	Enzymatic transformation
*Spirulina* sp.	Tetracycline	Adsorption
*Arunda donax*	Fluconazole	Bioodegradation and phytoaccumulation
*Chrysopogon zizanioides*	Ciprofloxacin and tetracycline	Phytouptake, phytodegradtion, detoxification
*Phragmites australis*	Ibuprofen, estrogens	Enzymatic transformation
*Arabidopsis thaliana*	Pharmaceuticals (cationic)	Transporter proteins

These species offer scalable options for remediating pharmaceuticals and heavy metals in wastewater and sludge.

Table 6 outlines the key advantages and limitations of bioremediation, emphasizing its eco-friendly nature, cost benefits, and applicability, while also addressing factors that can restrict its efficiency and field performance.

**Table 6:** Advantages and limitations of phytoremediation (Farraji et al., 2016).

Advantages	Limitations
Cost-effective compared to chemical/physical methods	Accumulated pollutants may enter food chain
Uses natural plant-microbe processes without harmful chemicals	Limited and sensitive to specific plant types, many of which are non-native
Targets a wide range of contaminants including metals, nutrients and organic matter	Low biomass yields mean repeated planting/harvesting is needed
Enhances microbial diversity and restores ecological balance	Hyperaccumulators often target only one pollutant type
Requires minimal infrastructure and is viable in rural/low-income areas	Use of chelating agents may cause secondary environmental issues
Scalable from lab trials to field-level deployment	Weather and season-dependent performance
Public-friendly, supports green esthetics and urban beautification	Safe disposal of contaminated biomass is necessary

## 6 Integrated treatment systems: synergistic approaches for HWW

HWW contains high levels of pharmaceuticals, disinfectants, antibiotic-resistant genes (ARGs), and pathogens, making it difficult to treat using single-method technologies. Integrated approaches that combine biological and physical–chemical systems have shown significantly improved performance for both pollutant removal and ARG reduction.

Standalone methods such as conventional activated sludge, constructed wetlands, or AOPs often fail to address the full spectrum of contaminants in HWW. Complex matrices require layered strategies that combine microbial degradation, plant uptake, and chemical oxidation. Integrated systems like MBR–AOP, CWs–PGP bacteria, and fungal–phyto consortia have demonstrated promising efficiencies by exploiting complementary mechanisms (Paulus et al., 2019; Riva et al., 2020).

### 6.1 Membrane bioreactor integration: the Pharmafilter case

A Dutch study demonstrated that advanced on-site systems like Pharmafilter, incorporating MBR technology, significantly outperform urban WWTPs in reducing antibiotics and ARGs. While conventional plants removed only 19–41% of key antibiotics, Pharmafilter eliminated 9 of 13 ARGs (including blaKPC, vanA, and intI1) and drastically lowered concentrations of ciprofloxacin (3,752 ng/L) and sulfamethoxazole (367 ng/L). In contrast, ozonation showed inconsistent ARG removal. The strong correlation between antibiotic and ARG concentrations emphasizes that reducing pharmaceutical loads curbs horizontal gene transfer by lowering selective pressure (Paulus et al., 2019).

### 6.2 CWs + PGP bacteria: plant–microbe synergy

Constructed wetlands (CWs) simulate natural ecosystems using macrophytes and substrate media to remove pollutants through sedimentation, sorption, and microbial degradation. Incorporating plant growth-promoting (PGP) bacteria such as *Pseudomonas* and *Burkholderia* enhances phytoremediation by improving nutrient uptake and plant tolerance under stress. These bacteria also boost the rhizospheric breakdown of antibiotics and organic micropollutants.

However, ARGs may spread via horizontal gene transfer in plant root zones, requiring careful species selection, temperature control, and real-time monitoring to mitigate risks. The balance between ecological benefit and resistance management is delicate but manageable (Riva et al., 2020).

### 6.3 CWs for emerging contaminants (ECs)

CWs are highly effective in removing emerging contaminants (ECs) like pharmaceuticals, personal care products (PPCPs), pesticides, hormones, PFAS, and microplastics. A Latin American review emphasized CWs as synergistic bio–phyto systems that utilize plant uptake, microbial activity, and substrate filtration. Removal rates up to 98% were reported for drugs like ibuprofen, caffeine, and triclosan, particularly when using plants such as *Eichhornia crassipes* and *Heliconia psittacorum* (Peña-Salamanca et al., 2025).

Table 7 summarizes the removal efficiencies achieved by various integrated treatment systems, highlighting how the combination of physical, chemical, and biological processes enhances the degradation of pharmaceutical contaminants.

**Table 7:** Removal efficiencies in integrated systems (Paulus et al., 2019; Peña-Salamanca et al., 2025; Riva et al., 2020).

System	Target pollutants	Removal efficiency
Pharmafilter (MBR-based)	Antibiotics, ARGs	19–99%
CW + PGP Bacteria	Nutrients, antibiotics, ARB	Moderate–High
Vertical Subsurface Flow (VSSF) Wetlands	Steroid hormones	55–100%
Hybrid CWs	PPCPs, hormones, PFAS, microplastics	High

### 6.4 Bacteria-assisted phytoremediation

To overcome phytoremediation’s limitations (e.g., plant toxicity, low uptake), researchers have employed endophytic bacteria microbes that live inside plant tissues and degrade pollutants using enzymes like oxygenases. Notable strains include *Pseudomonas*, *Bacillus*, and *Burkholderia*. These microbes not only degrade organic pollutants but also produce Indole Acetic Acid (IAA) and siderophores that promote plant health.

*Bacillus subtilis ZY16* is a strong candidate, supporting phytoextraction while reducing contaminant stress. Metagenomic tools now enable precise identification of such endophytes, though culture-based validations remain critical for field deployment (Karaś et al., 2021).

### 6.5 The WWTP contribution dilemma

Though hospitals contribute disproportionately to antibiotic and ARG loads, they often make up <1% of WWTP influent by volume. The persistence of ARGs in treated effluents has raised major concerns regarding WWTPs acting as reservoirs and amplifiers of antimicrobial resistance. Shotgun metagenomics in a pilot-scale hospital treatment plant revealed 264 unique ARGs across 22 resistance classes, with fluoroquinolone and tetracycline ARGs increasing post-treatment, indicating inadequate removal (Petrovich et al., 2020). In East China, hospital effluents from three treatment plants showed persistent *β*-lactam and quinolone ARGs, with ofloxacin poorly removed and pathogenic genera like *Acinetobacter* and *Klebsiella* surviving post-treatment due to sublethal chlorine exposure (Yao et al., 2021).

A study using 16S rRNA sequencing and qPCR found hospital sewage enriched in 12 of 14 ARG classes, yet post-treatment, ARG levels in WWTP effluents were similar to those of upstream urban sources. This suggests that integrated treatment at the source is more effective than relying on downstream dilution (Buelow et al., 2018). Similarly, a study in South Korea comparing domestic and HWW streams using 16S rRNA pyrosequencing found that hospital effluents contained more diverse and antibiotic-resistant microbial communities. Proteobacteria dominated hospital samples, while genera like *Zoogloea* thrived under antibiotic pressure. Despite >96% BOD removal, persistent compounds like n-hexane and copper showed limited removal, underscoring the inadequacy of conventional WWTPs and the value of continuous, metagenomics-based surveillance (Ahn and Choi, 2016). Another study in Norway found that although HWW had the highest concentration of resistant bacteria, community wastewater frequently harbored multidrug-resistant *E. coli*, indicating widespread resistance within the general population. The authors concluded that monitoring city-level wastewater provides a practical surveillance tool to assess antimicrobial resistance trends and inform public health strategies (Paulshus et al., 2019).

While integrated systems promise high removal rates, several challenges remain:

- Cost and Complexity: MBR–AOP and CW–bacteria systems require skilled management.
- Sludge and biomass handling: Especially with high pollutant loads.
- Site-specific customization: Not all hybrids work in all climates or settings.
- Policy and regulation: Especially for emerging contaminants and ARG surveillance.

Yet with proper monitoring, bioreactor control, and community-level adaptation, these systems represent the most future-ready approach for pharmaceutical wastewater treatment.

## 7 Challenges and research gaps

HWW presents a regulatory and environmental dilemma. Across countries, classification varies some treat it as domestic sewage, others as hazardous waste resulting in inconsistent monitoring and enforcement. In India, regulations depend on whether hospitals are connected to STPs, while the EU relies on general directives without hospital-specific provisions. Germany, China, Vietnam, and Italy all use distinct parameters like bed count or receiving water quality to define discharge standards (Carraro et al., 2018). A Greek healthcare facility reported significant gaps in waste management from improper segregation to ineffective incineration and revealed that pH-neutralized lab wastewater still exhibited high toxicity, with compounds like 5-fluorouracil and benzalkonium chloride escaping degradation (Tsakona et al., 2007). Similarly, a Turkish study at MFH1 hospital revealed that wastewater exhibited >90% cytotoxicity to kidney cells and strong mutagenicity via Ames testing, while also containing multidrug-resistant strains of *Enterobacteriaceae* and *Staphylococcus aureus*, underscoring the need for advanced monitoring and treatment strategies (Yilmaz et al., 2017).

The presence of antimicrobial-resistant bacteria (AMRB) and antibiotic resistance genes (ARGs) in HWW is a pressing concern. These pollutants originate from excreted drug residues, hospital surfaces, and biofilms, with common resistant strains including MRSA, VRE, and carbapenem-resistant *Pseudomonas* and *E. coli*. Resistance spreads via horizontal gene transfer, aided by mobile genetic elements such as plasmids and integrons (Werkneh and Islam, 2023).

Treatment technologies face serious limitations. CAS systems are energy-intensive and ineffective for many micropollutants. Even membrane bioreactors (MBRs) struggle with fouling and cost. Advanced methods like PAC, ozonation, and UV show promise, with PAC quickly adsorbing antibiotics and ozonation achieving >90% pharmaceutical removal. However, ozonation may form toxic byproducts, UV requires optimal water clarity, and chlorination may paradoxically increase AMRB levels. CWs and WSPs offer cost-effective phytoremediation but are not suitable for all conditions (Werkneh and Islam, 2023).

Scientific understanding of long-term contaminant behavior remains incomplete. Most studies focus on short-term lab results rather than field-scale trials. There’s limited data on pharmaceutical mixtures, ARG survival in biosolids, or the persistence of free-floating DNA post-treatment despite evidence that these contribute to resistance spread (Mehanni et al., 2023). Drug-resistant strains of *E. coli*, *Klebsiella pneumoniae*, and *Proteus mirabilis* were detected across all seven HWW treatment stages, including the final lagoon effluent, indicating persistent antimicrobial resistance (Kalaiselvi et al., 2016).

HWW harbors pathogens resistant to conventional treatment: *Salmonella*, *Klebsiella*, *Legionella*, *Candida*, *Aspergillus*, *Cryptosporidium*, and even SARS-CoV-2. Notably, *Cryptosporidium* oocysts resist chlorination and show high prevalence (18.9%), while fungal pathogens thrive in hot water systems. SARS-CoV-2 has been found in feces and aerosols, highlighting transmission risks during pandemics (Yuan and Pian, 2023).

Phytoremediation, though eco-friendly, is slow and limited in depth. It often requires multiple seasons to show results and cannot address complex pollutants deep in the soil profile (Lakhani et al., 2022). Further evidence from a comparative study of HWW treatment revealed that conventional systems often fail to remove resistant pathogens and mutagenic compounds. Some hospitals even showed increased post-treatment levels of bacteria like *Mycobacterium* and *Acinetobacter*, with total bacterial counts reaching 10^10^ cells/L. Ames testing confirmed mutagenicity in filtered effluent samples, indicating genotoxic risk. These findings reinforce the urgent need for hospital-specific treatment plants, ARG surveillance, and broader regulatory oversight to address both microbial and chemical threats (Timraz, 2016).

Regulatory inconsistency adds to the challenge. APIs have proliferated in the environment, with Indian rivers reporting concentrations above 14,000 μg/L. Many countries lack enforceable discharge limits, while reuse of inadequately treated effluents in agriculture remains widespread despite ecotoxic risks (N. A. Khan et al., 2020).

Healthcare waste management systems also lag. In Malaysia, waste is primarily incinerated or landfilled, releasing harmful byproducts. Though autoclaving and plasma pyrolysis offer cleaner alternatives, systemic issues like outdated infrastructure and poor enforcement persist (Ghasemi and Yusuff, 2016).

In summary, effective HWW management demands not only stricter regulations and ARG monitoring but also integrated systems capable of addressing emerging pollutants particularly pharmaceuticals, endocrine disruptors, heavy metals, and resistant microbial genes. Strengthening these measures is essential to limit the spread of resistance and the environmental persistence of toxic contaminants. By strengthening these measures, we are supporting SDG 6 (Clean Water and Sanitation), SDG 14 (Life Below Water), and SDG 15 (Life on Land).

## 8 Future perspectives and policy recommendations

### 8.1 Technological innovations and limitations

While technologies like membrane bioreactors (MBRs), AOPs, and constructed wetlands have demonstrated efficiency, persistent pollutants like erythromycin, carbamazepine, contrast agents, and ARGs remain challenging to eliminate (Verlicchi et al., 2015; Pariente et al., 2022). Future approaches must prioritize multi-barrier treatment trains, such as MBR + ozonation or fungal bioreactors followed by sand filtration, to maximize pharmaceutical and pathogen removal (Mir-Tutusaus et al., 2016). Solar-powered and decentralized systems could help reduce operational costs and energy demands, especially in low-resource settings (Verlicchi et al., 2015).

### 8.2 Emerging biotechnological and digital innovations

Emerging technologies including nanomaterials like TiO₂, CNTs, and iron oxide show strong potential for removing pharmaceuticals due to their high surface area and catalytic properties. However, issues related to aggregation, recovery, and environmental safety hinder their practical deployment. Research must focus on improving nanomaterial stability, retrieval, and low-toxicity profiles (Bagheri et al., 2016). Many other new techniques like bio-adsorption and electrochemical-biological methods are emerging as effective solutions for removing antidepressants from wastewater. These systems often use reactive oxygen species (ROS), generated through UV or combined AOPs, to accelerate degradation. For instance, 95% mineralization of amitriptyline was achieved within 360 min, and fluoxetine showed 86.14% removal when treated using an ozone–hydrogen peroxide system (Singh et al., 2022).

Synthetic biology and genetically modified plants offer exciting possibilities for phytoremediation. Natural hyperaccumulator plants tend to be slow-growing with limited pollutant uptake. Genetically engineered alternatives could improve biomass and contaminant removal efficiencies, though biosafety concerns and regulatory hesitancy persist (Lakhani et al., 2022).

Global collaboration is critical to tackle the shared challenge of pharmaceutical pollution. Public education campaigns, proper drug disposal systems, environmentally conscious prescription practices, and public–private partnerships can catalyze long-term solutions. Technologies like AI may assist in modeling pollutant behavior and optimizing treatment designs (Liu et al., 2024). The rapid expansion of data across various domains leaves conventional analytical techniques inadequate for managing complexity and size. This is where machine learning (ML) becomes essential. ML methods, especially artificial intelligence, are being used more and more in biological wastewater treatment. The primary aim of machine learning is to develop predictive models capable of making accurate predictions or judgements based on data-driven learning. A common use of ML, an advanced data analysis approach, is the detection of patterns or the formulation of predictions from extensive datasets produced by diverse scenarios. Artificial neural networks (ANN) can predict things like biochemical oxygen demand, total suspended solids, nitrogen, phosphorus, and chemical oxygen demand. However, they need a lot of computing power (Alprol et al., 2024; Ganthavee and Trzcinski, 2024).

### 8.3 Policy, regulation, and regional needs

A global review emphasized the need for on-site HWW treatment, stricter regulations, and improved worker safety protocols, particularly in regions lacking robust municipal infrastructure (Yan et al., 2020). Policy and regulation remain the most urgent areas for reform. While developed countries like the Netherlands, Germany, Switzerland, and France have implemented national plans including discharge limits, source separation, and “green” drug development many developing nations still treat HWW like ordinary domestic sewage (Chonova et al., 2018; Khan et al., 2020). For instance, Switzerland has prioritized centralized ozonation and activated carbon filtration in municipal WWTPs, while France mandates pharmaceutical take-back programs. Germany’s “Trace Substance Strategy” promotes green drug design, and the Netherlands enforces Water Quality Standards (WQS) for key pharmaceuticals in surface waters (Khasawneh and Palaniandy, 2021).

**Figure 3:**
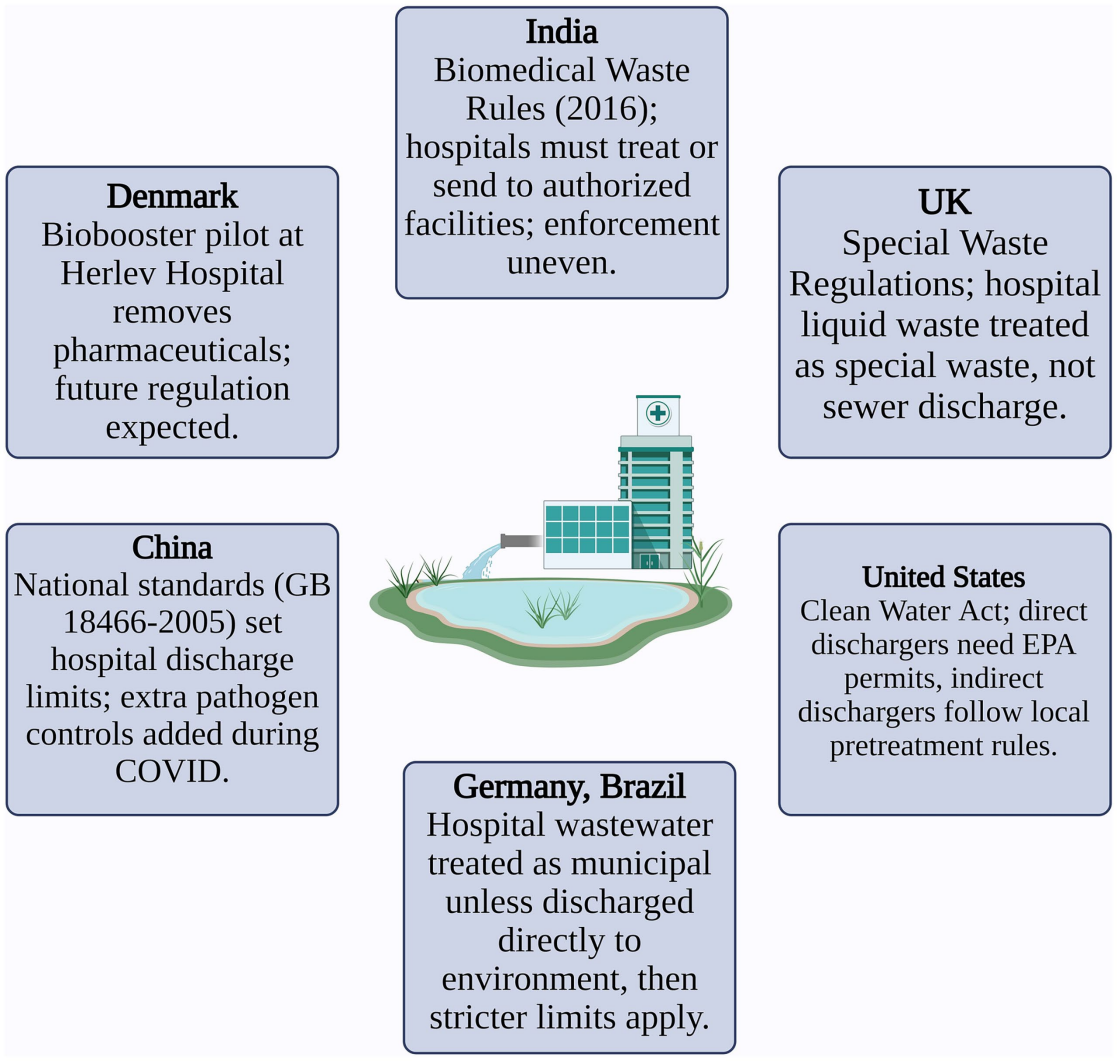
Global policy frameworks for HWW treatment.

Figure 3 illustrates global policy frameworks governing HWW treatment, showcasing the regulatory approaches, guidelines, and international standards implemented to manage pharmaceutical and pathogen contamination effectively.

India and other Asian countries, which face some of the highest pharmaceutical load emissions, urgently require legally enforceable guidelines tailored to HWW. Region-specific monitoring programs, supported by real-time sampling and risk quotient (RQ) analysis, are essential for identifying hotspots and evaluating long-term ecological risks (N. A. Khan et al., 2023).

To close existing gaps, future strategies must:

- Implement effluent discharge standards that are specific to hospitals and are designed to accommodate a broader spectrum of emerging pollutants, such as pharmaceuticals, endocrine-disrupting compounds, microplastics, and heavy metals.
- In addition to these chemical and biological contaminants, it is necessary to mandate the monitoring and reporting of ARGs.
- Encourage the development of treatment systems that are solar-supported, energy-efficient, and modular, and that can be customized to accommodate a wide range of contaminant profiles.
- Foster long-term, field-scale research that integrates sophisticated oxidation technologies, phytoremediation, and microbial processes to remove multiple contaminants.
- Ensure that pollutant management addresses interconnected human, environmental, and animal health risks by aligning national programs with One Health principles.

Only a combination of technological innovation, legislative enforcement, environmental stewardship, and community participation can ensure the safe and sustainable treatment of HWW.

## 9 Conclusion

HWW is a critical but under-regulated source of environmental contamination, laden with pharmaceuticals, antibiotic resistance genes (ARGs), and pathogens. Conventional treatment systems often fail to remove persistent pollutants like carbamazepine and sulfamethoxazole, contributing to antimicrobial resistance and ecological harm. Bioremediation and phytoremediation offer sustainable alternatives, with microbial consortia and rhizospheric plants demonstrating promising removal efficiencies. Integrated treatment frameworks combining membrane bioreactors, advanced oxidation processes, and hybrid bio-phyto systems emerge as the most effective solutions. However, gaps remain in regulation, real-world application, and long-term monitoring. The absence of standardized discharge limits, ARG surveillance, and toxicity assessments of pharmaceutical mixtures hinders broader implementation. To address these challenges, a multidisciplinary approach rooted in the One Health framework is essential. Investing in integrated technologies, strengthening region-specific policies, and promoting global collaboration can help mitigate the risks of hospital effluents and ensure a safer, more sustainable water future.
